# Comparative Radiological and Surgical Approaches in the Early Detection and Treatment of Colorectal Cancer: A Systematic Review

**DOI:** 10.7759/cureus.106930

**Published:** 2026-04-13

**Authors:** Varsha Mary Khalkho, Sunil Kumar Yadav, Manisha Mandwe, Deepshikha, M. Pavunraj, Alka Dave

**Affiliations:** 1 Department of Radiology, School of Medical Sciences, Sri Satya Sai University of Technology and Medical Sciences, Sehore, IND; 2 Department of General Surgery, Pandit Bhagwat Dayal Sharma Post Graduate Institute of Medical Sciences, Rohtak, IND; 3 Department of Radiology, Jain Diagnostic Centre, Jabalpur, IND; 4 Department of Medical Oncology, Homi Bhabha Cancer Hospital and Research Centre, Muzaffarpur, IND; 5 Department of Zoology, Vivekananda College - Autonomous and Residential, Tiruvedakam, IND; 6 Department of Anesthesiology, Dr. N. D. Desai Faculty of Medical Sciences and Research, Dharmsinh Desai University, Nadiad, IND

**Keywords:** adenoma detection rate, colorectal cancer, magnetic resonance imaging, surgical oncology, total mesorectal excision

## Abstract

One of the biggest causes of cancer mortality is still colorectal cancer (CRC), and outcomes depend heavily on timely detection, accurate staging, and curative-intent intervention, having been among the top five causes of cancer-related death globally since 1990 and, as of 2023, the leading cause of cancer-related death in individuals under 50. Evidence comparing radiological and surgical strategies remains fragmented across screening, staging, and treatment pathways, limiting direct translation into unified clinical workflows. This review aimed to compare the diagnostic and clinical utility of major imaging modalities with outcomes of surgical approaches used in early-stage CRC. A systematic search of electronic databases was conducted using MeSH and free-text terms, restricted to English-language human studies published between 2015 and 2025. Randomised trials and observational comparative studies were included, with data extracted on imaging accuracy, staging concordance, perioperative outcomes, recurrence, and survival, followed by narrative synthesis due to heterogeneity. Key outcomes showed CT colonography as a feasible screening alternative with cost-effectiveness dependent on participation, pelvic MRI as central for rectal staging and restaging, and PET-based techniques contributing mainly to surveillance and metastatic assessment. Surgical evidence supported minimally invasive colectomy and organ-preserving rectal pathways in selected patients, while emphasising morbidity risks and recurrence trade-offs. These findings support multidisciplinary integration of imaging-guided stratification with tailored surgical planning and biomarker-informed escalation or de-escalation. Limitations included variable protocols and inconsistent endpoint reporting. Precision imaging combined with appropriate surgery remains the most actionable strategy for improving early CRC outcomes.

## Introduction and background

In most regions of the world, colorectal cancer (CRC) continues to account for a substantial proportion of cancer-related morbidity and mortality, making it a major public health concern. Screening has contributed significantly to reducing disease burden, with colonoscopy remaining the reference standard for detection and prevention through adenoma removal [1]. While CRC encompasses malignancies of the colon and rectum, it is important to recognise that these represent distinct clinical entities with differences in staging, imaging requirements, and surgical management, necessitating tailored diagnostic and therapeutic approaches [2].

In early-stage disease, surgery remains the cornerstone of curative treatment [3]. However, the choice of surgical strategy is highly dependent on accurate preoperative imaging, which defines tumour location, depth of invasion, involvement of adjacent structures, and resectability [4,5]. This is particularly critical in rectal cancer, where pelvic anatomy and margin status directly influence decisions regarding local excision, total mesorectal excision, or neoadjuvant treatment strategies [6]. In contrast, colon cancer staging and operative planning rely more heavily on cross-sectional imaging for assessment of locoregional and distant disease [7,8].

Imaging plays a central role across the CRC care pathway. For initial staging, modalities such as computed tomography (CT) are routinely used to evaluate systemic disease, while pelvic magnetic resonance imaging (MRI) is the standard for local staging in rectal cancer, enabling assessment of tumour depth, mesorectal fascia involvement, and high-risk features associated with recurrence [9,10]. In addition, imaging findings directly inform treatment stratification, including the selection of patients for neoadjuvant therapies, organ-preserving strategies, or immediate surgical intervention [11].

At the population level, CRC presents with varying stages at diagnosis, with a substantial proportion of patients identified at regional or advanced stages, highlighting the need for improved early detection and optimised staging pathways [12]. This variability reinforces the importance of integrating accurate imaging with appropriate surgical planning to improve outcomes in potentially curable disease [13].

Despite advances in both radiological techniques and surgical approaches, there remains a lack of clear synthesis regarding how different imaging modalities influence surgical decision-making and clinical outcomes in early-stage CRC, particularly when considering the distinct pathways of colon and rectal cancers. Existing literature often evaluates these components independently, limiting the ability to understand their combined impact on patient management.

Objectives of the review

This review aims to systematically compare radiological and surgical approaches used in the early detection, staging, and treatment of CRC, with a primary focus on early-stage disease and clear differentiation between colon and rectal cancer pathways. It evaluates the diagnostic performance and clinical utility of key imaging modalities alongside outcomes of major surgical strategies. In addition, it examines how imaging-guided surgical planning influences treatment selection, recurrence risk, and clinical outcomes, while identifying gaps in current evidence to inform future research and clinical practice.

## Review

Methodology

Search Strategy

A comprehensive literature search was conducted using PubMed/MEDLINE, Scopus, and Web of Science to identify studies evaluating radiological and surgical approaches in the early detection, staging, and treatment of CRC. The final search was performed in December 2025. Controlled vocabulary (MeSH terms) and free-text keywords related to colorectal neoplasms, diagnostic imaging (CT, MRI, PET/CT, endorectal ultrasound), and surgical techniques (colectomy, local excision, total mesorectal excision) were combined using Boolean operators.

A representative PubMed search strategy included the following: (“colorectal cancer” OR “colorectal neoplasms”) AND (“imaging” OR “CT” OR “MRI” OR “PET/CT” OR “endorectal ultrasound”) AND (“surgery” OR “colectomy” OR “local excision” OR “total mesorectal excision”).

The search was limited to studies published in English, involving human participants, between 2015 and 2025. Reference lists of eligible studies were also screened to identify additional relevant articles. The full search strategy was adapted for each database using combinations of controlled vocabulary and free-text terms related to CRC, imaging modalities, and surgical techniques. Filters were applied for human studies, English language, and publication years between 2015 and 2025.

Eligibility Criteria

Inclusion criteria: Studies were included if they evaluated at least one radiological modality relevant to detection or staging, and/or one surgical intervention relevant to curative treatment of CRC, and reported measurable clinical outcomes. Eligible study designs included randomised controlled trials, prospective or retrospective observational studies, and comparative cohort studies involving adult patients.

Outcomes of interest included diagnostic accuracy (e.g., sensitivity, specificity), staging performance, surgical outcomes, recurrence, survival, and complication rates. Colon and rectal cancers were treated as distinct entities due to differences in staging, imaging, and surgical management, and findings were interpreted accordingly.

Eligibility was restricted to studies involving early-stage and locally advanced (non-metastatic) disease relevant to curative-intent management. Studies focusing exclusively on metastatic disease, surveillance-only cohorts, or feasibility analyses without clinical outcome measures were excluded.

Exclusion criteria: Excluded studies included reviews, conference abstracts without full text, case reports, non-English publications, paediatric or animal studies, in vitro studies, and studies lacking extractable or clinically relevant outcomes. Studies focusing solely on non-surgical therapies without imaging relevance were also excluded.

Study Selection

After the removal of duplicates, citations were managed manually without the use of reference management software. Titles and abstracts were screened independently by two reviewers, followed by a full-text review of potentially eligible studies. Disagreements were resolved through discussion and consensus. Data extraction was also performed independently by two reviewers using a predefined framework, with discrepancies resolved through discussion and consensus.

Data Extraction

A standardised data extraction approach was used. Extracted variables included study characteristics (author, year, country, design), patient demographics, tumour location (colon vs rectum), imaging modality, surgical technique, comparator groups, and reported outcomes.

Radiological outcomes included lesion detection rates, diagnostic accuracy, staging concordance, and modality-specific limitations. Surgical outcomes included operative time, blood loss, margin status, lymph node yield, complication rates, conversion rates, length of stay, recurrence, and survival.

Quality Assessment

Methodological quality was assessed using tools appropriate to the study design. Randomised controlled trials were evaluated for randomisation, allocation procedures, follow-up completeness, and outcome reporting. Observational studies were assessed for baseline comparability, confounding control, and consistency of outcome measurement.

Risk of Bias Assessment

Risk of bias was assessed at the study level using appropriate tools based on study design. Randomised controlled trials were evaluated using the Cochrane Risk of Bias 2 (RoB 2) tool, while non-randomised studies were assessed using the ROBINS-I tool [14]. Domains assessed included selection bias, performance bias, detection bias, attrition bias, and reporting bias. For observational studies, additional consideration was given to confounding and baseline imbalance. Disagreements in risk of bias assessment were resolved through consensus between reviewers.

Data Synthesis

Due to substantial heterogeneity in study populations, imaging protocols, staging criteria, surgical techniques, and outcome measures, a quantitative meta-analysis was not planned. Instead, findings were synthesised narratively and presented in structured tables to facilitate comparison across imaging modalities and surgical strategies. Where appropriate, outcomes were grouped by modality and intervention type to support interpretation of patterns.

This review was conducted in accordance with PRISMA guidelines, and the study selection process is summarised in the PRISMA flow diagram.

Results

Study Selection

**Figure 1:**
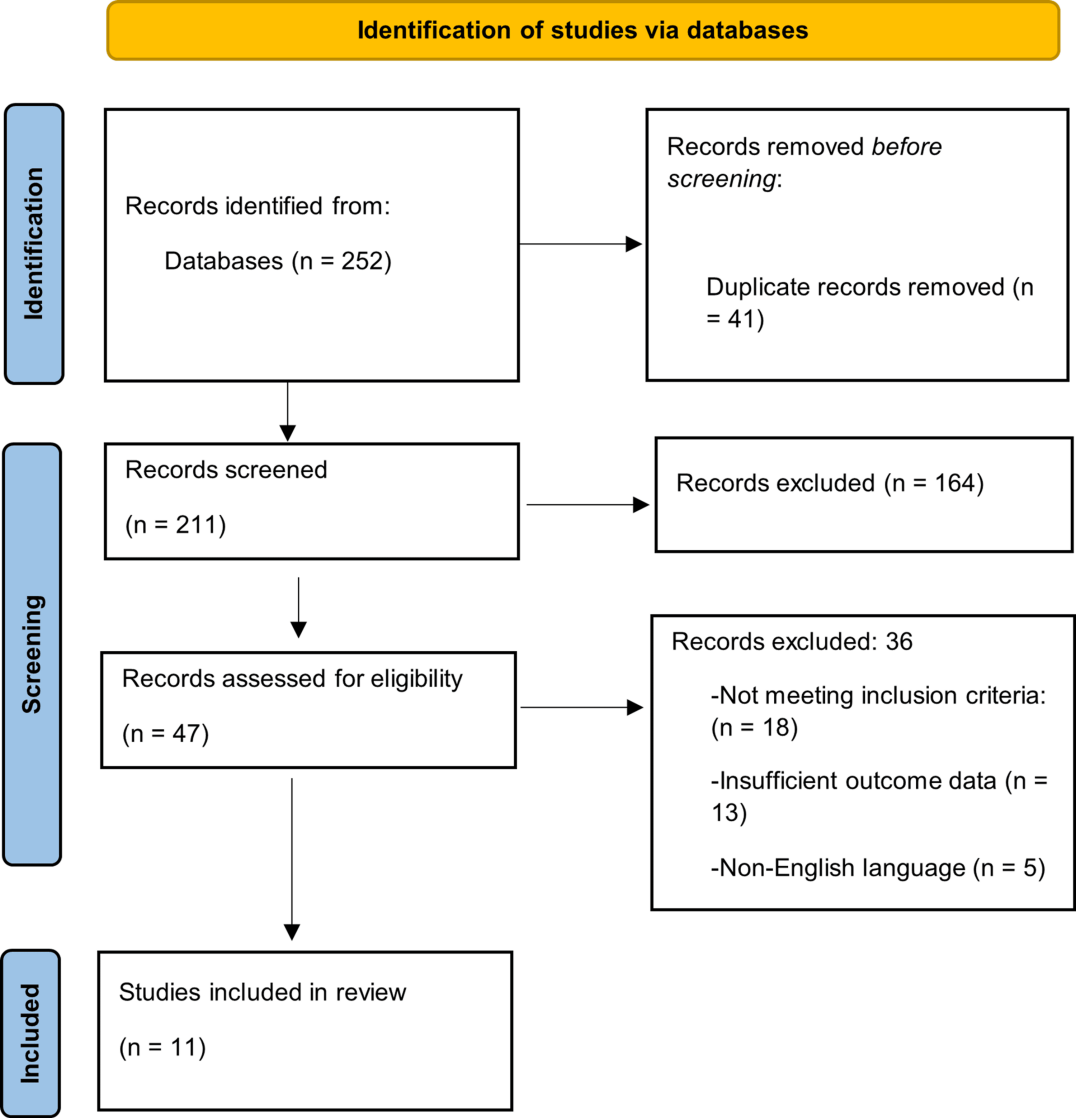
PRISMA flowchart The figure was created using Microsoft Word (Microsoft® Corp., Redmond, WA). Reference: [15]

The initial database search identified 252 records. After removing 41 duplicates, 211 unique records remained for title and abstract screening. Of these, 164 records were excluded because they were not relevant to the study, leaving 47 articles for full-text assessment. Following full-text review, 36 articles were excluded, primarily due to failure to meet the inclusion criteria (n = 18) or insufficient outcome data (n = 13). A smaller number were excluded because they were non-English publications (n = 5). As a result, 11 studies were included in the final review. No prior protocol was registered for this review. Disagreements between reviewers during study selection were resolved through discussion and consensus. Figure 1 presents the study selection process in accordance with the PRISMA [15] flow diagram.

Characteristics of Included Studies

The studies included were a combination of randomised studies, prospective observational studies, and retrospective cohort studies published in 2015-2025. The population studied was heterogeneous and included screening cohorts, patients with the early stage of CRC, and patients with the locally advanced rectal cancer who had undergone multimodal therapy. In the literature, there was a variation in the anatomical focus, as some studies assessed colon cancer as a whole, whereas others assessed rectal cancer specifically, because imaging has a great impact on the planning of surgery. Sample sizes varied between small institutional cohorts and big multicenter trial populations. Reported outcomes were heterogeneous, although they usually comprised diagnostic accuracy measures of radiologic modalities and perioperative, oncologic, and recurrence-related outcomes of surgical modalities. The properties and the most important comparative results of the studies included in Table 1 are the evaluation of radiological and surgical methods in the early detection and treatment of CRC.

**Table 1:** Study characteristics and key findings of included studies cCR: clinical complete response; CRC: colorectal cancer; CRT: chemoradiotherapy; CT: computed tomography; DFS: disease-free survival; FDG: fluorodeoxyglucose; iCR: incomplete response; MRI: magnetic resonance imaging; nCR: near-complete response; OPRA: organ preservation in patients with rectal adenocarcinoma; OS: overall survival; OSNA: one-step nucleic acid amplification; PET/CT: positron emission tomography/computed tomography; PET/MRI: positron emission tomography/magnetic resonance imaging; RCT: randomised controlled trial; RT: radiotherapy; TNT: total neoadjuvant therapy

Study	Domain	Modality and surgical procedure	Study design and population	Comparator	Main outcomes assessed	Key findings
Rutegård et al. [10]	Radiology (hybrid staging)	PET/MRI and PET/CT hybrid imaging (RECTOPET study)	Prospective descriptive/feasibility study; rectal cancer	PET/MRI vs PET/CT	Imaging description, initial performance observations	Reported early observations on hybrid imaging approaches for rectal cancer using PET/MRI and PET/CT
Dijkstra et al. [16]	Surgery + radiology-guided multimodal care	RAPIDO trial: short-course RT + chemotherapy + surgery (TNT)	Randomised trial follow-up: locally advanced rectal cancer	TNT vs long-course CRT + surgery	Distant metastasis, locoregional recurrence, OS	TNT improved systemic control but showed higher locoregional recurrence at longer follow-up; OS remained similar
Williams et al. [17]	Radiology (restaging/organ preservation)	Restaging pelvic MRI after TNT in rectal cancer	Trial-based analysis: rectal cancer patients after TNT	MRI response categories (cCR vs nCR vs iCR)	Organ preservation, local regrowth, DFS, OS	MRI response categories predicted organ preservation, local regrowth, and survival
Sobhani et al. [18]	Radiology (surveillance)	6-monthly 18FDG PET/CT monitoring in CRC	Open-label multicentre randomised trial	PET/CT monitoring vs standard follow-up	Recurrence detection, monitoring effectiveness, and downstream management	PET/CT was evaluated as a structured surveillance tool using 6-monthly monitoring protocols
Kennecke et al. [19]	Surgery + multimodal (organ preservation)	Neoadjuvant chemotherapy → excision → observation (NEO trial)	Phase II trial; early rectal cancer	Protocol-based pathway	Feasibility, organ preservation outcomes	Evaluated neoadjuvant chemotherapy followed by excision and observation as an organ-preservation strategy
van der Meulen et al. [20]	Radiology (screening economics)	CT colonography screening	Cost-effectiveness modelling study (population screening)	Colonoscopy vs CT colonography	Cost-effectiveness, participation effects, screening trade-offs	CT colonography cost-effectiveness depended on participation and screening cost assumptions
Yeung et al. [21]	Surgery + intraoperative imaging	Fluorescence imaging + OSNA lymph node assessment during laparoscopic CRC surgery	Observational/feasibility surgical study	Standard nodal assessment vs fluorescence + OSNA	Intraoperative node identification, nodal analysis feasibility	Demonstrated feasibility of fluorescence imaging combined with rapid OSNA for nodal identification and assessment
Song et al. [22]	Surgery (minimally invasive)	Single-incision laparoscopic surgery (SILS) for CRC	Single-centre open-label non-inferiority RCT	SILS vs conventional laparoscopic surgery (CLS)	5-year DFS, OS, recurrence patterns, incisional hernia	5-year DFS and OS were similar between SILS and conventional laparoscopy
Bao et al. [23]	Surgery (postoperative complication impact)	Low anterior resection (mid-low rectal cancer)	Extended follow-up of randomised trial	Leak vs no leak	Long-term oncologic outcomes	Anastomotic leak was associated with long-term oncologic outcome implications.
Sylla et al. [24]	Surgery (rectal cancer technique)	Transanal total mesorectal excision (taTME)	Multicenter phase II trial; rectal cancer	None (single-arm)	30-day morbidity, anastomotic outcomes	Early morbidity included urinary retention and anastomotic complications extending beyond 30 days
Hansdotter et al. [25]	Surveillance imaging + surgical salvage	COLOFOL recurrence pattern analysis after curative surgery	Prospective cohort within RCT population; stages II-III CRC	High-intensity vs low-intensity CT follow-up	Recurrence timing/site, resectability, OS	Mapped recurrence patterns and evaluated which recurrences were amenable to curative-intent treatment

Radiological Approaches in Early Detection of Colorectal Cancer

Radiological modalities have been playing the main role in screening and detection routes. CT colonography was also often considered as an alternative screening method of the minimally invasive type, especially when patients could not complete the colonoscopy procedure. The largest lesion was generally reported to be detected best with a higher detection rate of the large adenoma and invasive lesions than the small or flat lesions. In screening situations, detection using imaging was frequently referred to in conjunction with referral protocols to confirmatory colonoscopy and biopsy. Where possible, radiological screening and endoscopic methods were compared, and it was demonstrated that colonoscopy is the more invasive modality, but it offers the major advantage of direct mucosal visualisation with immediate polypectomy, making it the reference standard for adenoma detection and early CRC assessment. By contrast, CT colonography is less invasive and may also reveal incidental extracolonic findings requiring further evaluation, but its diagnostic performance is more limited for smaller or flat lesions, and it may also lead to false-positive interpretations, such as normal mucosal folds mimicking polyps, and positive findings still require follow-up colonoscopy for histologic confirmation and removal. Table 2 demonstrates the evidence synthesised on the radiological modalities in the screening, staging/restaging, and surveillance of CRC.

**Table 2:** Comparative synthesis of radiological approaches in early detection, staging, and surveillance of colorectal cancer CRC: colorectal cancer; CT: computed tomography; DFS: disease-free survival; MRI: magnetic resonance imaging; OS: overall survival; PET/CT: positron emission tomography/computed tomography; PET/MRI: positron emission tomography/magnetic resonance imaging; TNT: total neoadjuvant therapy

Radiological modality	Clinical role in CRC pathway	Main outcomes assessed in the included studies	Synthesis of findings	References
CT colonography	Screening alternative to colonoscopy; population-level screening evaluation	Cost-effectiveness, participation effects, screening efficiency	CT colonography was evaluated as a screening strategy with emphasis on cost-effectiveness relative to colonoscopy, with outcomes influenced by participation rates and screening cost assumptions.	van der Meulen et al. [20]
Pelvic MRI (restaging)	Restaging after TNT; response stratification for organ preservation (watch-and-wait vs surgery)	DFS, OS, local regrowth, prediction of residual disease	Restaging MRI response categories were associated with long-term outcomes, supporting MRI-based stratification for organ preservation and recurrence risk after neoadjuvant therapy.	Williams et al. [17]
PET/CT (surveillance)	Monitoring and detection of recurrence after CRC treatment	Surveillance effectiveness, recurrence monitoring	PET/CT was assessed as a structured surveillance tool using 6-monthly monitoring protocols in a randomised trial framework.	Sobhani et al. [18]
PET/MRI and PET/CT hybrid imaging	Staging and post-treatment imaging feasibility in rectal cancer	Imaging feasibility and initial observations	Hybrid imaging approaches were reported with early descriptive findings, supporting their role as emerging techniques in rectal cancer imaging evaluation.	Rutegård et al. [10]

Radiological Approaches in Staging and Preoperative Assessment

The staging and surgical planning process involved imaging, particularly in rectal cancer. For initial staging, clear distinctions were made between colon and rectal cancer pathways: in rectal cancer, local staging was performed using pelvic MRI to evaluate tumour depth, mesorectal fascia involvement, and local recurrence risk factors, whereas in colon cancer, staging primarily relied on CT imaging for assessment of local and distant disease. The local staging in rectal cancer was always provided using MRI on the pelvis to evaluate tumour depth, mesorectal fascia involvement, and local recurrence risk factors, as MRI serves as the cornerstone for rectal cancer staging and directly influences treatment decisions, including the feasibility of transanal resection, the need for total mesorectal excision, and selection for neoadjuvant therapies such as total neoadjuvant therapy (TNT), watch-and-wait strategies, or other non-surgical approaches.

For systemic staging, CT scans of the chest, abdomen, and pelvis were routinely used across both colon and rectal cancers to evaluate distant metastases. In the context of restaging, particularly following neoadjuvant therapy in rectal cancer, MRI and PET/CT were utilised to assess treatment response and guide further management decisions. The use of PET/CT was mainly in the restaging, recurrence, and metastatic analysis, particularly in cases where conventional imaging data were inconclusive.

For surveillance, PET/CT and CT imaging were employed selectively to detect recurrence, especially in high-risk or equivocal cases. In the selection of studies that used endorectal ultrasound, the application occurred in early rectal tumours, especially in determining candidacy for local excision. In the research, the correlation with operative pathology outcomes, margin status, and recurrence patterns was often used as a measure of staging performance.

Surgical Approaches for Early-Stage Colorectal Cancer

The surgical treatment methods incorporated conventional oncologic resections for colon cancer, including segmental colectomy with lymphadenectomy, and total mesorectal excision for rectal cancer, with the choice of surgical approach primarily determined by preoperative imaging findings, including tumour location, depth of invasion, and involvement of adjacent structures as outlined above. Minimally invasive colectomy was widely studied in terms of outcomes compared to those of open surgery, where applicable.

In the case of early rectal cancer, local excision surgery was reported as an organ-conserving option, including transanal excision, transanal endoscopic surgery, and endoscopic minimally invasive surgery in carefully selected patients, with eligibility for these approaches dependent on imaging-based staging and confirmation of limited tumour invasion.

The perioperative outcomes reported in the studies included operative duration, blood loss, conversion to open surgery, length of hospital stay, and complication rates. Functional outcomes, including bowel, urinary, and sexual dysfunction, were also commonly discussed in rectal cancer cohorts because of the morbidity associated with radical pelvic surgery. Table 3 presents the evidence on surgical strategies and pathways within combined radiology-guided management in CRC.

**Table 3:** Comparative synthesis of surgical approaches and integrated radiology-guided management in CRC COLOFOL: colon cancer follow-up trial; CRC: colorectal cancer; CT: computed tomography; DFS: disease-free survival; NEO: neoadjuvant chemotherapy followed by excision and observation; OS: overall survival; OSNA: one-step nucleic acid amplification; RAPIDO: rectal cancer and preoperative induction therapy followed by dedicated operation; RCT: randomised controlled trial; TME: total mesorectal excision

Surgical approach	Clinical indication	Study design focus in included studies	Synthesis of findings
Total neoadjuvant therapy (TNT) + surgery (RAPIDO)	Locally advanced rectal cancer	Long-term oncologic outcomes in RCT follow-up	TNT improved systemic control but raised concerns about locoregional recurrence at longer follow-up, emphasising the need for careful imaging-based selection and surgical planning.
Single-incision laparoscopic surgery (SILS)	Curative CRC resection in selected patients	Non-inferiority RCT with long-term follow-up	SILS demonstrated comparable long-term DFS and OS to conventional laparoscopic surgery when performed under standardised oncologic principles.
Transanal total mesorectal excision (taTME)	Rectal cancer requiring TME; technique-focused outcomes	Multicentre phase II evaluation	TME outcomes emphasised postoperative morbidity patterns, including urinary retention and anastomotic complications extending beyond early postoperative periods.
Neoadjuvant chemotherapy → excision → observation (NEO pathway)	Early rectal cancer with intent for organ preservation	Phase II trial outcomes	The pathway supported the feasibility of organ preservation strategies using neoadjuvant therapy followed by excision and structured observation.
Anastomotic leak impact after low anterior resection	Mid-low rectal cancer postoperative complication	Extended follow-up of RCT	Anastomotic leak was associated with long-term oncologic outcome implications, supporting its importance as a prognostic and quality indicator in rectal surgery.
Intraoperative fluorescence imaging + OSNA	Laparoscopic CRC surgery (nodal assessment enhancement)	Feasibility/technical study	Fluorescence imaging combined with rapid OSNA was used to support intraoperative lymph node identification and analysis workflows.
CT-based surveillance and recurrence resectability (COLOFOL analysis)	Stages II-III CRC after curative surgery	Recurrence mapping within the RCT cohort	Recurrence patterns and their resectability were evaluated, supporting the role of structured follow-up in identifying potentially curable recurrences.

Comparative Radiology-Surgery Findings

**Figure 2:**
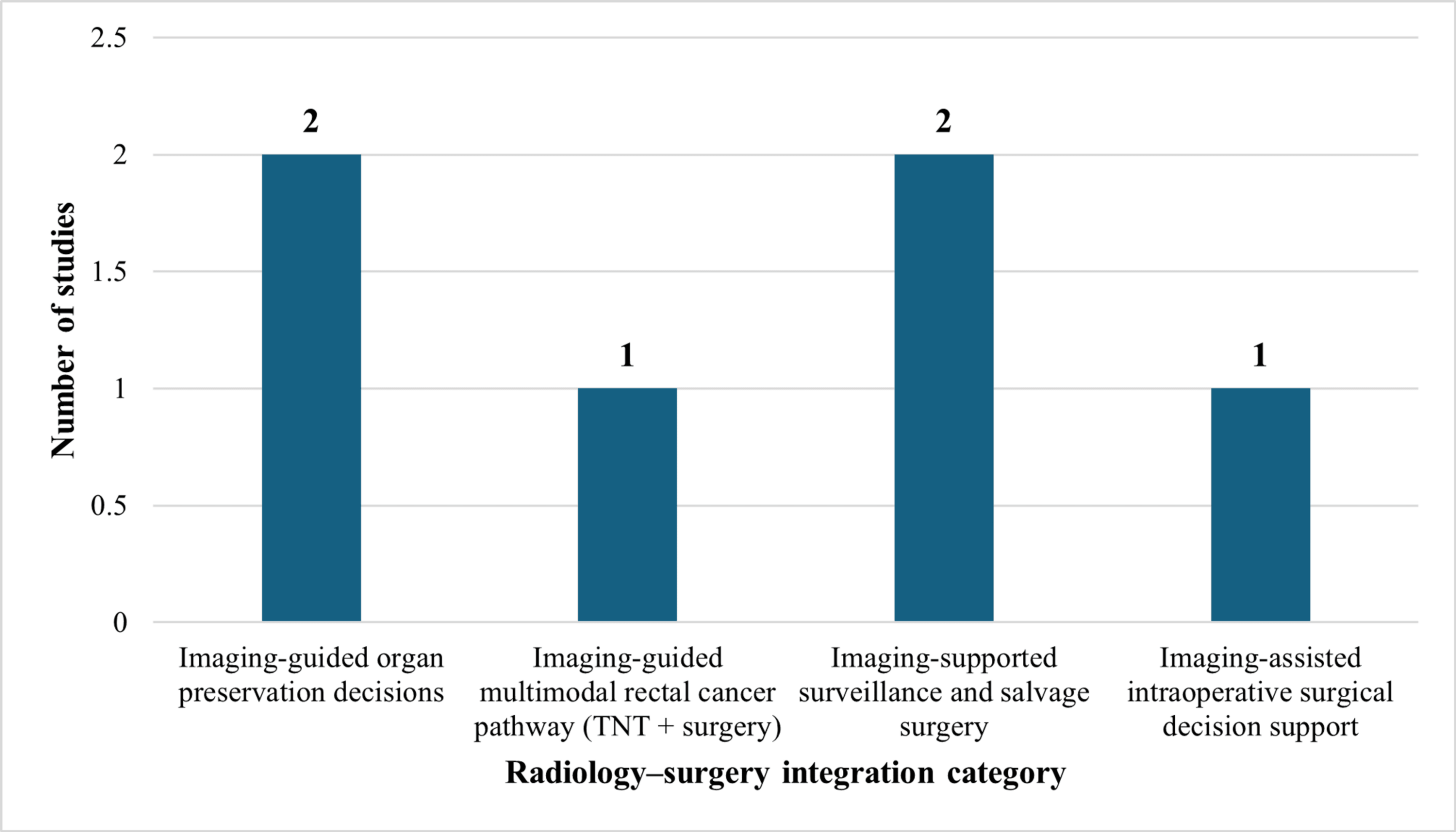
Comparative radiology-surgery integration themes across included studies The figure was created using Microsoft Excel (Microsoft® Corp., Redmond, WA). TNT: total neoadjuvant therapy

Included studies were also found to implicate radiological findings in surgical decision-making. In rectal cancer, imaging-based staging was found to affect the choice of local excision versus radical resection, and in neoadjuvant treatment before surgery. The assessment of circumferential resection margin risk with the MRI was recurrently characterised as being a predictor of the strategy employed in the surgery and the possibility of the need for multimodal therapy. In both early and progressive disease, imaging was also utilised in defining resectability and in identifying whether treatment was curative or palliative. The concepts of multidisciplinary review processes were often mentioned as one of the main factors in combining radiological assessment with the process of operative planning and selection of suitable surgical interventions. Figure 2 indicates the distribution of included studies by topic of radiology-surgery integration, with the majority of the included studies on surveillance/salvage and organ preservation.

Oncologic Outcomes Across Included Studies

Some of the oncologic outcomes that were reported in studies were margin, lymph node yield, local recurrence, distant recurrence, disease-free survival, and overall survival. In the case of rectal cancer surgery, the involvement of the circumferential resection margin was one of the primary endpoints, which is commonly correlated with risk stratification through MRI. In the case of colectomy studies, lymph node harvest sufficiency and R0 resection were more often used as predictors of oncologic completeness. Liver, lung, and local pelvic recurrence, and the time of recurrence detection were the common outcomes of recurrence being reported. The reported variation in survival outcomes across studies makes them less directly comparable, as this is due to differences in the duration of follow-up and definitions of endpoints.

Post-treatment Surveillance, Recurrence Detection, and Resectability

Recurrence was also highly detected by the use of follow-up imaging to inform the decision on salvage treatment. A review of recurrence rates also found that high recurrence rates were identified in the first three years following curative resection, and this has highlighted the clinical significance of organised surveillance regimens. Follow-up CT was most commonly employed in the detection of liver and lung metastases, whereas MRI and PET/CT were sparingly used based on the suspicion of recurrence and local availability of the resource. Resectability of recurrence was frequently published; some studies reported that multidisciplinary tumour board review enhanced the possibility of potentially curative therapy, including metastasectomy, ablative modalities, or resection of local recurrences, which were isolated.

Complications, Functional Outcomes, and Quality of Life

The profiles of complications differed according to the strategies of surgery and depended on the location of the tumour and the type of surgery. The results of colectomy were anastomotic leakage, postoperative infection, ileus, and cardiopulmonary problems. Surgical outcome in rectal cancer was associated with an increased incidence of functional morbidity, including bowel dysfunction, urinary dysfunction, and sexual dysfunction, which were commonly reported. There were organ-preserving approaches like local excision or watch-and-wait protocols, which were claimed to have the purpose of minimising surgical morbidity but were necessitated by the intensive surveillance and cautious patient selection. Harms related to radiology were mostly addressed in connection with radiation (in particular with screening and surveillance using CT), false-positive results, as well as incidental extracolonic findings resulting in further studies.

Evidence Quality and Risk of Bias Summary

The design quality of the included research varied. Randomised trials tended to have higher internal validity but were constrained by strict eligibility criteria that reduced generalizability. A substantial proportion of the evidence was derived from observational and comparative studies, which were more susceptible to selection bias, confounding, and incomplete outcome reporting. Across the dataset, imaging protocols, staging criteria, and surgical techniques were not reported uniformly, limiting comparability between studies. The most commonly identified risks of bias included baseline imbalances between groups, inadequate control of confounders, and variability in outcome reporting. Interpretation of findings was therefore undertaken with consideration of study quality and risk of bias, with greater weight assigned to studies demonstrating higher methodological rigour and transparent reporting.

The level of risk of bias across the included studies is presented in Table 4, which encompasses a range of study designs, including randomised controlled trials, observational studies, feasibility studies, modelling analyses, and single-arm trials; accordingly, appropriate risk of bias assessment tools were applied based on study design rather than exclusively using the Cochrane RoB 2 tool [14].

**Table 4:** Risk of bias assessment of included studies RCT: randomised controlled trial

Study	Study design	Selection bias	Performance bias	Detection bias	Overall risk of bias
Rutegård et al. [10]	Prospective feasibility/observational hybrid imaging study	Moderate	Low	Moderate	Moderate
Dijkstra et al. [16]	Randomised controlled trial (5-year follow-up)	Low	Moderate	Low	Low
Williams et al. [17]	Secondary analysis of a prospective trial cohort	Low	Moderate	Moderate	Moderate
Sobhani et al. [18]	Open-label multicentre randomised trial	Low	Moderate	Moderate	Moderate
Kennecke et al. [19]	Phase II trial (protocol-based)	Moderate	Moderate	Moderate	Moderate
van der Meulen et al. [20]	Cost-effectiveness modelling study	Moderate	Low	Moderate	Moderate
Yeung et al. [21]	Observational feasibility study	Moderate	Low	Moderate	Moderate
Song et al. [22]	Single-centre open-label randomised non-inferiority trial	Low	Moderate	Moderate	Moderate
Bao et al. [23]	Extended follow-up of randomised trial	Low	Moderate	Low	Low
Sylla et al. [24]	Multicentre phase II trial (single arm)	Moderate	Moderate	Moderate	Moderate
Hansdotter et al. [25]	Prospective cohort within RCT population	Moderate	Moderate	Moderate	Moderate

Discussion

CRC management has evolved with advances in screening, imaging, and surgical techniques, with increasing emphasis on accurate staging and imaging-guided treatment planning in early-stage disease. This review highlights that optimal outcomes are closely linked to the integration of diagnostic imaging with appropriately selected surgical strategies, although heterogeneity in study design and outcome reporting continues to limit direct comparability.

In the context of early detection, improved lesion detection through advanced colonoscopic techniques has demonstrated enhanced diagnostic yield; however, the generalisability of these findings remains dependent on variations in baseline adenoma detection rates, operator expertise, and population characteristics [1]. These findings reinforce that screening performance metrics should be interpreted within the context of local practice rather than universally extrapolated. Importantly, imaging modalities should not be viewed as replacements for colonoscopy but as complementary tools, particularly for staging and preoperative assessment, whereas colonoscopy remains essential for direct visualisation, biopsy, and polypectomy.

Imaging plays a central role in guiding clinical decision-making [26]. In rectal cancer, pelvic MRI provides critical information on tumour depth, mesorectal fascia involvement, and high-risk features, directly influencing decisions regarding neoadjuvant therapy, organ preservation strategies, and surgical approach [27]. In colon cancer, CT imaging remains essential for assessing disease extent and resectability. These modality-specific roles highlight the importance of distinguishing between colon and rectal cancer pathways when interpreting imaging findings.

The integration of imaging with surgical planning is particularly relevant in rectal cancer, where accurate staging determines eligibility for local excision, total mesorectal excision, or non-operative management strategies [28]. Similarly, appropriate radiological staging in colon cancer supports operative planning and identification of patients suitable for curative resection. These findings emphasise that imaging is not merely diagnostic but plays a pivotal role in treatment selection and risk stratification.

Although emerging biomarkers such as circulating tumour DNA may offer additional value in postoperative risk stratification, their role remains adjunctive and does not replace the central importance of imaging in guiding surgical decision-making. Variability in assay standardisation and implementation further limits their routine integration into clinical pathways at present [6].

Overall, the evidence supports a model of CRC management in which imaging and surgery are closely integrated, with modality-specific strengths informing tailored treatment strategies. However, the heterogeneity of the available literature and the predominance of non-randomised data necessitate cautious interpretation of these findings and highlight the need for more standardised, comparative research.

Limitations and Future Recommendations

The heterogeneity in the study designs, patient and outcome-reporting in radiological and surgical domains, limits this review because it restricted direct comparability and could not provide a powerful quantitative synthesis. There could have been differences in imaging regimens, definitions of staging, and levels of surgical skill and expertise, which could have contributed to the reported accuracy of diagnosis and oncologic outcomes. The publication bias and English-language restriction might have decreased the inclusion of the relevant evidence in this area, and some of the included trials did possess selective inclusion criteria to restrict their generalizability to standard clinical groups.

Future studies should emphasise multicenter comparative designs that incorporate standardised imaging protocols, clearly defined surgical pathways, and uniform outcome measures to improve comparability and methodological consistency. The longitudinal studies are required to explain the impact of imaging-based organ preservation interventions on functional outcomes and recurrence in the long term. Surveillance supported by biomarkers and adjuvant decision models based on ctDNA use is to be considered in various populations in order to deal with equity and feasibility of implementation. Culturally specific interventions, such as the use of culturally-sensitive methods of delivery, and the measurement of real-world adherence, cost-effectiveness, and patient-centred outcomes, should be included in screening interventions.

## Conclusions

According to this systematic review, early detection strategies, radiological staging, and surgical decision-making lead to the greatest improvement in the outcomes of CRC when they are combined into a synchronised clinical process. Radiological modalities are supported as critical to detection, assessment of response, and surveillance of recurrence, whereas surgery is the ultimate treatment measure of localised disease, and minimal invasive and organ-sparing techniques can be safely applied to patients in a well-selected group. The results of the study answer the main questions based on which the CT methods and MRI methods demonstrate the most clinically relevant information on screening and rectal staging, the imaging-guided planning has a direct impact on margin risk and treatment choice, and surgical practice leads to good oncologic results when it is supported by an accurate preoperative evaluation. The evidence can be interpreted to mean that imaging findings remain central to clinical decision-making in CRC, particularly in guiding surgical planning and treatment stratification, with clear distinctions required between colon and rectal cancer due to differences in staging, imaging modalities, and operative approaches. However, the study design heterogeneity, imaging protocols and inconsistent reporting caused decreased direct comparability and prevented a consistent quantitative synthesis. Standards-based multicentre trials, endpoints harmonisation between radiology and surgical outcomes and studies on implementation of screening adherence and equity should be considered in future work. The general idea is that accurate imaging and appropriately tailored surgical approaches play a central role in the management of early-stage CRC, with emerging biomarker-driven strategies offering additional potential to refine treatment decisions; however, these conclusions should be interpreted with caution, given the heterogeneity of the included studies and the narrative nature of this review.
